# Preparation and Barrier Performance of Layer-Modified Soil-Stripping/Cassava Starch Composite Films

**DOI:** 10.3390/polym12071611

**Published:** 2020-07-20

**Authors:** Lijie Huang, Xiaoxue Han, Haobin Chen, Shuxiang An, Hanyu Zhao, Hao Xu, Chongxing Huang, Shuangfei Wang, Yang Liu

**Affiliations:** 1 College of Light Industry and Food Engineering, Guangxi University, Nanning 530004, China; 1916391010@st.gxu.edu.cn (X.H.); 1916391001@st.gxu.edu.cn (H.C.); 1716391001@st.gxu.edu.cn (S.A.); 1816301036@st.gxu.edu.cn (H.Z.); 1816391033@st.gxu.edu.cn (H.X.); 2 Guangxi Key Laboratory of Clean Pulp & Papermaking and Pollution Control, Nanning 530004, China; huangcx@gxu.edu.cn (C.H.); wangsf@gxu.edu.cn (S.W.); xiaobai@gxu.edu.cn (Y.L.)

**Keywords:** montmorillonite, intercalation reaction, starch film, barrier mechanism

## Abstract

In this study, we investigated the barrier properties of a montmorillonite-reinforced biomass material, starch. Organically modified montmorillonite materials were prepared from natural montmorillonite by reacting it with dodecyl trimethyl ammonium chloride, dodecyl dimethyl benzyl ammonium chloride or octadecyl trimethyl ammonium chloride under ultrasonic conditions. The composite starch films incorporated with these organically modified montmorillonite samples were characterized by scanning electron microscopy, transmission electron microscopy, infrared spectroscopy and X-ray diffraction. The results showed that the introduction of montmorillonite decreases the transmittance of the composite film by 10% in the visible region and significantly inhibits UV-light transmittance. The decomposition temperature of the composite film ranges from 200 to 500 °C, with a weight loss rate of 80%. The distance between the montmorillonite layers increases from 0.14 nm in the non-magnetized state to 1.49 nm after magnetization. The oxygen permeability of the starch film modified by organic montmorillonite (0.067 cm^3^/m^2^·d) is lower than that of the montmorillonite starch film without magnetization (0.097cm^3^/m^2^·d). The oxygen barrier capacity is close to zero. Particularly in the ordered magnetic montmorillonite starch composite film, the oxygen barrier ability is the best. Therefore, modified montmorillonite could serve as an excellent reinforcing agent for cassava starch films and effectively improve the oxygen barrier performance of the films.

## 1. Introduction

Starch is a naturally occurring polymer. A starch solution is typically prepared by heating starch with water. Because of its good film-forming properties, a stable starch film can be formed by casting it onto a smooth plane from a solution [1]. The film-formation abilities of amylose and amylopectin in starch-based films are different. Compared to the films prepared from starch with a high amylopectin content, those prepared from starch with a high amylose content are more easily formed with more favorable ductility and barrier properties. Sun et al. prepared cornstarch films using silica as an enhancer [2]. By exploring the properties of silica as well as corn starch flowing into the film, the optimum preparation process for nanosilica starch films was determined. Basiakand et al. stratified rapeseed oil by the three-layer pressing of starch and prepared starch films by pouring the starch after the stratification reaction. The absorption of water by the starch film was markedly reduced and the starch absorbed fat from the oil, resulting in decreased tensile strength and a decreased contact angle of the starch film with an increase in the amount of rapeseed oil [3].

Montmorillonite is a silicate substance with a typical lamellar structure; it has a 2:1 hierarchical structure and bond-type structures such as Al–O and Si–O chain structures in the interior. The introduction of organic modifiers can increase the distance between the montmorillonite lamella and increase the transmission path for molecules, thereby improving the barrier properties [4,5]. Because of its strong surface activity, montmorillonite can undergo inorganic-ion exchange with silver ions; further, the organic modification of montmorillonite with cetyltrimethylammonium bromide can result in a nanocomposite film with antibacterial properties and improved barrier properties, providing film size selectivity for different types of materials [6,7]. Dong and co-workers prepared composite films of polyhexalactone/montmorillonite/chitosan, and reported that the moisture resistance and oxygen resistance of the composites improved with the addition of montmorillonite [8]. Liu et al. added montmorillonite to polyvinyl alcohol (PVA) via solution intercalation to improve the oxygen resistance of packaging materials based on a PVA composite, thus reducing the energy consumption of duck egg packaging, as well as increasing the shelf life and reducing the loss of salted duck eggs during transport [9]. Although many researchers have studied montmorillonite as a reinforcement agent for composite films, the barrier properties of starch-based films with organically modified montmorillonite introduced via intercalation under ultrasonic conditions have not been studied.

In recent years, starch-based films have been used in packaging. However, they have not found wider use in other commercial and consumer goods, owing to their poor mechanical and barrier properties [10]. Although the chemical properties of montmorillonite have allowed its use in surfactants, the improvement in the barrier properties of polymer materials incorporated with montmorillonite has not been widely explored. An excellent barrier effect can be achieved with the minimal addition of montmorillonite, which aligns with the concepts of green packaging [11]. By using economic and practical materials from a wide range of sources, one can prepare packaging materials wherein the biomass materials can degrade, the material performance is improved and thus, local economic crops can be diversified by promoting green and environment-friendly packaging science and technology.

Herein, we studied the barrier properties of a montmorillonite-reinforced biomass material, starch. We prepared different organically modified montmorillonite samples (hereafter referred to as organic montmorillonite) by reacting montmorillonite with different organic modifiers (amines) at different dosages for different reaction times and used each of them as a reinforcing agent to improve the barrier properties of starch films. Furthermore, by magnetizing the organic montmorillonite using an iron chloride solution, we increased the distance between the montmorillonite layers and thus extended the passage of small gas molecules in the composite material to enhance the barrier mechanism of the composite film. The composite film can be used in the fields of packaging, agricultural moisture retention, everyday preservation and medicine and is hence of great significance.

## 2. Materials and Methods 

### 2.1. Materials

The materials used include montmorillonite (pharmaceutical grade, Shanghai Aladdin Biochemical Technology Co., Ltd., Shanghai, China), dodecyl trimethylammonium chloride (DTAC; 99%, Shanghai Aladdin Biochemical Technology Co., Ltd.), dodecyl dimethyl benzyl chloride ammonium chloride (DDBAC; 98%, Shanghai Aladdin Biochemical Technology Co., Ltd.), octadecyl trimethyl ammonium chloride (STAC; 98%, Shanghai Aladdin Biochemical Technology Co., Ltd.), anhydrous ethanol (analytical grade, Xilong Science Co., Ltd., Shantou, China), silver nitrate (analytical grade, Tianjin Damao Chemical Reagent Factory, Tianjin, China), phosphotungstic acid (analytical grade, Tianjin Damao Chemical Reagent Factory), potassium bromide (spectral grade, Shanghai Aladdin Biochemical Technology Co., Ltd.), natural tapioca starch (industrial grade, Guangxi Cenxi Triangle Starch Co., Ltd., Cenxi, China), glycerin (98%, Shanghai Aladdin Biochemical Technology Co., Ltd.), ferrous chloride (analytical grade, Tianjin Damao Chemical Reagent Factory), ferric chloride (analytical grade, Tianjin Damao Chemical Reagent Factory) and sodium hydroxide (analytical grade, Tianjin Damao Chemical Reagent Factory).

### 2.2. Material Preparation

#### 2.2.1. Preparation of Organic Montmorillonite 

Montmorillonite was dried in a 50 °C blast-drying oven for 24 h, then cooled in a drying dish to obtain dry montmorillonite. The dry montmorillonite (30 g) was weighed and suspended in distilled water (600 mL), then ultrasonically dispersed at room temperature (25–27 °C) for 1 h and finally magnetically stirred for 12 h (500 r/min) to obtain a montmorillonite suspension. Next, DTAC (6 g) was added to the montmorillonite suspension, stirred at room temperature for 30 min and ultrasonically dispersed for 1 h. The mixture was stirred in a water bath at 80 °C for 3 h, allowed to cool to room temperature and dispersed again for 1 h. The suspension was then centrifuged (7000 rpm, 10 min), following which the precipitate was washed with 50% aqueous ethanol to remove Cl^−^ (until no precipitate was detected with the use of a 0.2 M AgNO_3_ solution) and was finally dried in a blast-drying oven at 50 °C for 24 h. The dried montmorillonite was ground and passed through a 120 mesh sieve to obtain organic montmorillonite.

DTAC (3 g each) was added to three montmorillonite suspensions, which were then reacted under different ultrasonic conditions for 0, 1 and 2 h. Thereafter, the suspensions underwent water-bath cooling, ultrasonication, centrifugation, washing, drying, sieving and other operations to obtain samples labeled 0-1 DTAC, 1-1 DTAC and 2-1 DTAC, respectively. Further, 3, 6 and 9 g of DTAC; 6 g of DDBAC; and 6 g of STAC were separately added to five other montmorillonite suspensions, which were reacted under ultrasonic conditions for 1 h to obtain samples labeled 1-1 DTAC, 1-2 DTAC, 1-3 DTAC, 1-2 DDBAC and 1-2 STAC, respectively.

#### 2.2.2. Preparation of Magnetized Organic Montmorillonite

Organic montmorillonite (2 g, 1-2 DTAC) was added to a three-necked flask with distilled water (200 mL) and the mixture was stirred for 12 h to form a suspension. This suspension was then dispersed ultrasonically for 30 min. Thereafter, the device was vented with argon to exhaust the air in the flask. Subsequently, FeCl_3_ (0.01 mol) and FeCl_2_ (0.005 mol) were dissolved in 100 mL of water. The iron solution was quickly poured into the Ar-filled flask and stirred, followed by the addition of a 0.2 M NaOH solution to adjust the pH of the mixed suspension to 11. This suspension was stirred for 1 h at 80 °C in a water bath, after which the stirring was stopped and the suspension was ultrasonicated for 30 min. The resulting product was magnetically separated out as a black precipitate, which was washed multiple times with distilled water and dried at 50 °C for 24 h in a vacuum oven to obtain magnetic organic montmorillonite.

#### 2.2.3. Preparation of Cassava Starch/Modified Montmorillonite Composite Films

The organic montmorillonite (0.5 g) was dispersed in water (50 mL), sonicated for 1 h and magnetically stirred for 12 h to form a suspension. Subsequently, tapioca starch (5 g), glycerol (1.5 g) and water (100 mL) were added to the organic montmorillonite suspension and the resulting mixture was stirred under heating in a water bath at 80 °C for 30 min. The heating was then stopped while stirring was continued at room temperature [12]. Ultrasonication (60 Hz) was performed for 30 min to obtain a starch/montmorillonite film-forming suspension. Subsequently, 48 g of the film-forming suspension was placed in a 12 cm × 12 cm square plastic petri dish and dried in a drying oven at 50 °C for 10 h to obtain an organic montmorillonite/starch composite film.

A three-necked flask was purged with Ar gas to create an inert atmosphere in the flask. The modified montmorillonite was weighed out at 0.25–1 g (5, 10, 15 and 20 wt %) and added into the three-necked flask containing 50 mL of distilled water, then sonicated for 1 h and mechanically stirred for 12 h to form a magnetic organic montmorillonite suspension. Subsequently, tapioca starch (5 g), glycerin (1.5 g) and water (100 mL) were added to the montmorillonite suspension and the resulting mixture was mixed well. This mixture was then heated to 80 °C and mechanically stirred for 30 min, followed by stirring while cooling to room temperature. Additional ultrasonication for 30 min yielded a magnetic starch/montmorillonite film-forming suspension. Further, 48 g of the film-forming suspension was taken in a 12 cm × 12 cm square plastic Petri dish. To obtain a disordered material, the film-forming suspension was dried in a drying box at 50 °C for 10 h, while an ordered material was obtained by drying the film-forming suspension naturally in air under parallel magnetic induction lines produced by a strong magnet for 24 h, to obtain magnetic organic montmorillonite/starch films [13,14].

## 3. Testing and Characterization

### 3.1. Apparent Morphological Characterization 

The apparent morphology and size of the organic montmorillonite and magnetic organic montmorillonite were characterized by transmission electron microscopy (TEM; Hitachi HT7700, Hitachi Hitech Company) at an acceleration voltage of 80 kV. Prior to TEM, a droplet of the suspension of organic montmorillonite was loaded onto a copper mesh and dried.

The microscopic morphology of the organic montmorillonite and organic montmorillonite/starch composite films were characterized by scanning electron microscopy (SEM; F16502, Phinom, The Netherlands) at the test voltage of 10 kV. The sample used for observation was placed on the conductive adhesive of the electron microscope loading table and then treated with a gold spray.

### 3.2. Barrier Performance 

The moisture permeability of the magnetic organic montmorillonite/starch composite films and organic montmorillonite/starch composite films with different contents (5, 10, 15 and 20 wt %) of organic montmorillonite was determined according to the GB/T1037-1988 standard using a water vapor transmittance tester (TSY-T1; Jinan Languang Electromechanical Technology Center).

The prepared film-forming liquid (60 g) was placed in a circular plastic Petri dish of diameter 15 cm. The ordered and disordered magnetic starch films were dried by the same method to test their oxygen permeation performance.

### 3.3. Mechanical Properties 

The tensile strength and elongation at break of the magnetic montmorillonite/starch composite films and montmorillonite/starch composite films with different amounts of montmorillonite were measured and analyzed using an electronic universal material testing machine (INSTRON, Boston, MA, USA). The ASTM-D882-02(2002) test method was used, with samples 100 mm × 10 mm in size, an initial fixture distance of 50 mm and stretching speed of 1 mm/s.

### 3.4. Infrared Spectroscopy

The magnetized and non-magnetized organic montmorillonite powders were ground to 200 mesh (a grain for filtering) and characterized by Fourier-transform infrared (Bruker, Karlsruhe, Germany) spectroscopy. The scanning wavelength range was 400–4000 cm^–1^ and the resolution was 4 cm^−1^.

### 3.5. X-ray Diffraction (XRD)

The magnetized and non-magnetized organic montmorillonite powders dried at 50 °C for 2 h were characterized by X-ray diffraction (XRD) (Rigaku, Tokyo, Japan.). The scanning range was 2θ = 3–10° and the scanning speed was 5°/min. The spacing in montmorillonite was calculated according to the following formula [15]:

(1) 2dsinθ=λ(1),

where *d* is the montmorillonite spacing in nanometers, *θ* is the diffraction angle of the characteristic crystal plane in degrees and *λ* is the X-ray wavelength in nanometers.

### 3.6. Thermal Stability 

The thermal weight loss of the montmorillonite samples prepared under different ultrasonication durations using different modifiers at different dosages was analyzed by synchronous thermogravimetric analysis (TA Instruments, New Castle, DE, USA), to analyze the thermal stability of each sample. TGA was conducted under a N_2_ gas flow of 40 mL/min at the heating rate of 10 °C/min in the temperature range of 30–60 °C.

### 3.7. Contact Angle

The hydrophobicity of the montmorillonite/starch composite films was studied with the Kruss contact angle measurement (PHOENOM, Eindhoven, The Netherlands). The montmorillonite/starch film was cut into a slender specimen (25 mm × 20 mm) and the specimen was tightly attached to a glass slide. The slide containing the sample was placed on the carrier table of the measuring instrument and the water droplet size was set to 3 μL.

## 4. Results

### 4.1. Apparent Morphological Analysis

**Figure 1:**
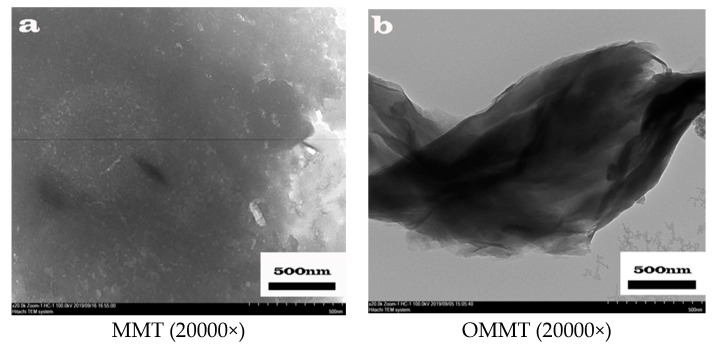

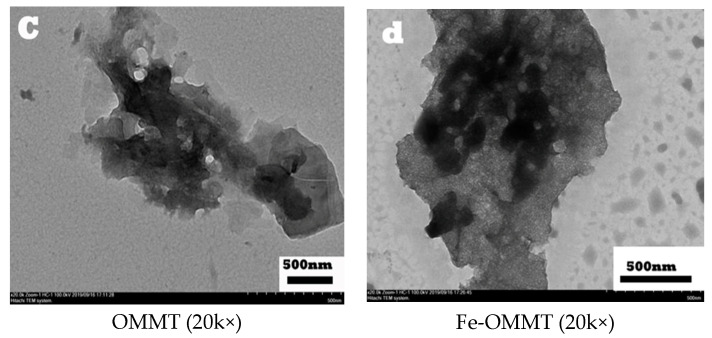
Transmission electron micrographs of montmorillonite and magnetic montmorillonite: (**a**) montmorillonite (MMT), (**b**) organically modified montmorillonite (OMMT), (**c**) magnetic montmorillonite (OMMT), (**d**) magnetic organic montmorillonite (Fe-OMMT).

Figure 1a,b show the TEM images of the non-modified montmorillonite and one of the organically modified montmorillonite samples prepared under 1 h of ultrasonication, respectively. Figure 1a shows that the non-modified montmorillonite is agglomerated; it has a dense layer spacing and the distance between each layer cannot be distinguished. Figure 1b, on the other hand, indicates a distinct reduction in the layer agglomeration of the organic montmorillonite; the layered structure and interlayer gap of the montmorillonite can be clearly observed. These results indicate that the organic modifier was successfully intercalated between the montmorillonite interlayers and they increased the interlayer spacing of the montmorillonite, thus effectively elongating the effective paths for small molecule transport through the material [16]. Figure 1c,d show the TEM images of magnetic organic montmorillonite. The organically modified montmorillonite has a clear lamellar structure. Before the magnetization reaction, the interlayer distance of montmorillonite is already known. After the introduction of magnetic montmorillonite, due to its magnetic properties, one can clearly see the arrangement of organic carbon chains in the interlayer of montmorillonite. The magnetic substance is wrapped on the carbon chain, which together support the gap between the layers of montmorillonite and increase it too.

**Figure 2:**
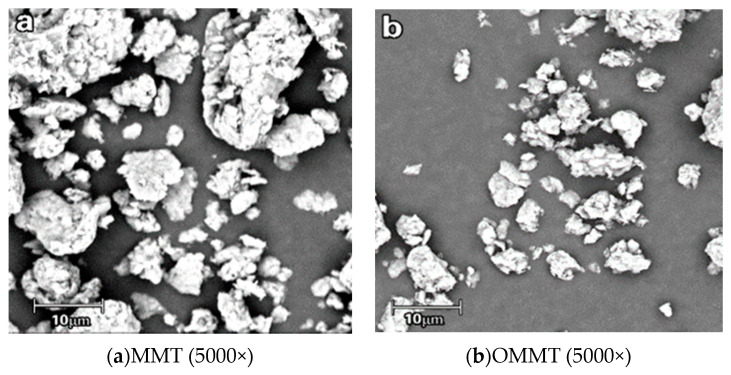

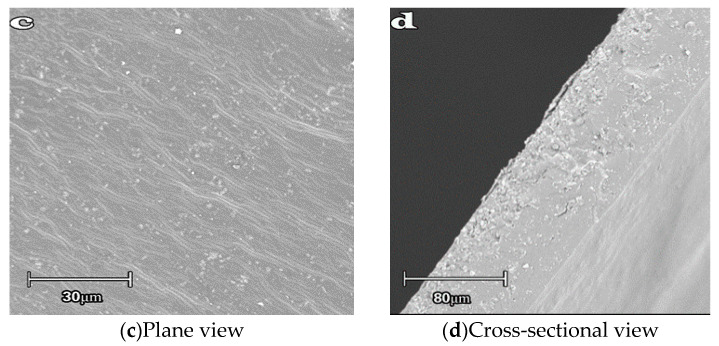
Scanning electron microscopy (SEM) images of montmorillonite and organic montmorillonite/starch films: (**a**) montmorillonite (MMT), (**b**) organically modified montmorillonite (OMMT), (**c**) plane view of OMMT/starch film and (**d**) cross-section of OMMT/starch film.

Figure 2 shows the SEM images of unmodified montmorillonite (Figure 2a) and one of the organically modified montmorillonite samples (Figure 2b) prepared with 1 h of ultrasonication. Owing to its strong cation-exchange capacity, montmorillonite easily agglomerates to form particles in the case of a natural dispersion, as shown in Figure 2a [17]. After organic modification, the molecular agglomerates become smaller, indicating that the modifier could effectively enter the interlayer structure and react with the cations of the montmorillonite, thus reducing its agglomeration. The plane-view SEM image of the organic montmorillonite/starch film (Figure 2c) shows that the organic montmorillonite has a three-layered structure that can support a stable layered structure after binding with starch [18]. The organic montmorillonite supports the increasing or decreasing distance of the montmorillonite layers and the starch can be fully combined with montmorillonite. Furthermore, the network supported by the three-layer sheet structure of montmorillonite can be clearly seen to facilitate full contact between the montmorillonite and starch, aiding the formation of a smooth and dense organic film. As montmorillonite particles are relatively small, they are evenly distributed within the composite film and the thickness of the resulting film is not uniform. The composite film is flat over a large area with no starch particles, indicating that after a 30 min water-bath reaction, the starch had completely gelatinized to form a film-forming liquid, resulting in the film morphology being similar to that of a starch film without montmorillonite. Figure 2d shows a SEM image of the cross-section of the starch film, which is uniform and evenly dispersed with organic montmorillonite [19].

### 4.2. Analysis of the Barrier Performance

**Figure 3:**
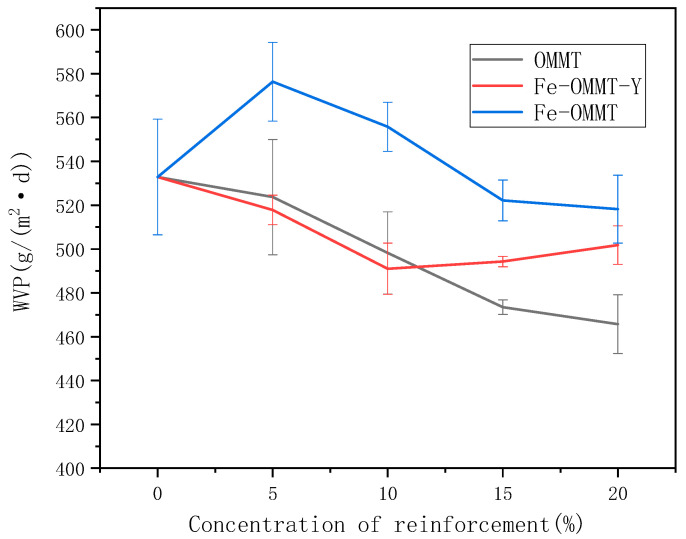
Moisture permeabilities of starch films with different amounts of the reinforcement agent.

Figure 3 shows the relationship between the concentration of the reinforcing agent (magnetized and non-magnetized organic montmorillonite) in the composite starch films and their moisture permeability. The hygroscopic properties of the non-magnetized montmorillonite/starch films did not improve with an increase in the content of montmorillonite because of the good barrier properties of montmorillonite. In contrast, the moisture permeability of the composite film with magnetic montmorillonite decreased slightly as its amount was increased. From 576.38 g/(m^3^∙24 h) to 518.1933 g/(m^3^∙24 h). The water absorption capability of the hydroxyl groups in the magnetic montmorillonite allows the montmorillonite to act as a barrier to water vapor in the composite film, because of the change in its Fe–O bond. However, the composite films with both, non-magnetized and magnetized organic montmorillonite, have a relatively small range of moisture permeability, because starch itself is more sensitive to water vapor [20,21].

**Figure 4:**
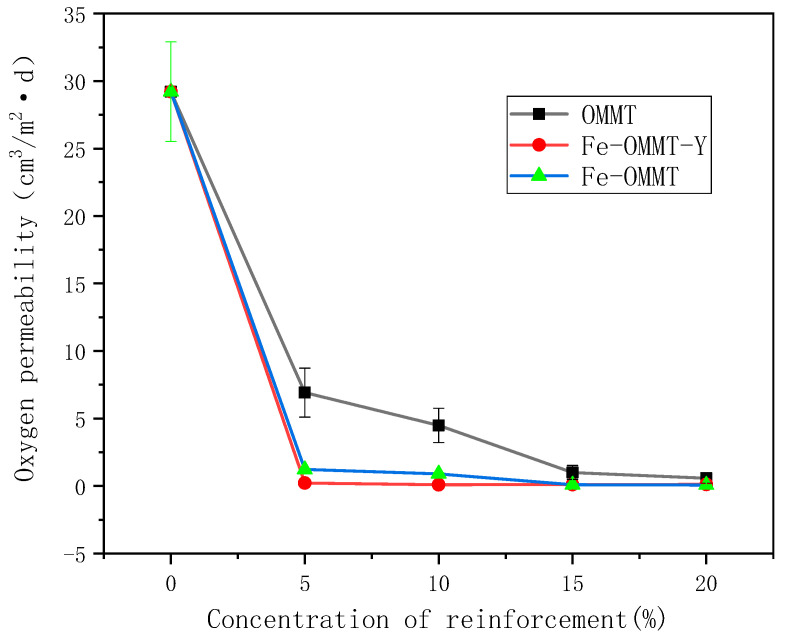
Oxygen permeabilities of starch films with different amounts of the reinforcement agent.

Figure 4 shows the influence of the montmorillonite content in the composite starch films on their oxygen permeability. With increasing amount of montmorillonite added to the montmorillonite starch film without magnetization modification, the oxygen permeability of the composite film decreases from 6.917 to 0.56 cm^3^/m^2^·d, because it is known that montmorillonite has a lamellar structure. With an increase in the distance between the montmorillonite layers, the path for organic materials (such as small gas molecules) is elongated and therefore, the modified montmorillonite with increased interlayer spacing enhances the barrier properties of these films [22]. The oxygen permeability of the organic montmorillonite-modified starch film is lower (0.067 cm^3^/m^2^·d) than that of the montmorillonite starch film without magnetization modification (0.097 cm^3^/m^2^·d). The oxygen barrier capacity is close to zero. The increased interlayer spacing of montmorillonite after organic modification can widen the path for molecular transmission due to the support of carbon chains. However, because the starch film particles are modified with organic montmorillonite, the formation pathway and increase in the interlayer distance are small. As a result, although the barrier performance of the composite is markedly improved, it does not reach the ideal barrier capability of approximately zero. The oxygen permeability of the ordered magnetic montmorillonite starch composite film increases with the addition amount, from 0.23 to 0.113 cm^3^/m^2^·d and the effect is stable. Owing to the high quality of the magnetic montmorillonite and its magnetic effect, a montmorillonite/starch film with layers oriented in a single direction (layer orientation) is formed with the longest possible path. Thus, the best barrier properties of montmorillonite/starch films are obtained with ordered composite films in this study. Compared with the disordered starch film, the ordered starch film can achieve a constant gas-barrier effect faster and more stably and the introduction of Fe can effectively improve the barrier properties of the montmorillonite/starch films.

### 4.3. Analysis of Mechanical Properties

**Figure 5:**
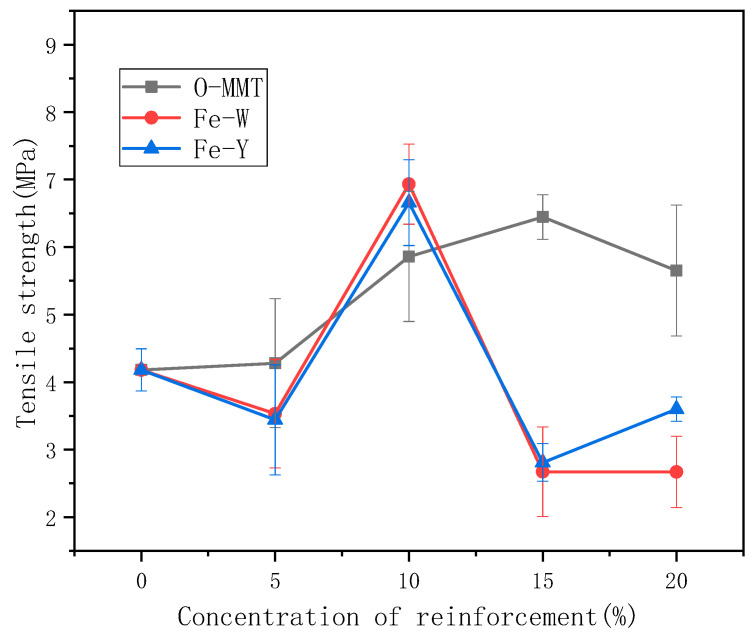
Tensile strengths of starch films with different amounts of the reinforcement agent.

Figure 5 shows the change in the tensile strength of the montmorillonite/starch composite films with different amounts of modified montmorillonite. The tensile strength of the non-magnetized composite film increases as the amount of added montmorillonite increases. The modified montmorillonite can be used as a filler to fill the starch molecules. The film-forming liquid is uniform, in which starch and montmorillonite are tightly linked through chemical bonding; this enables improvement in the tensile strength of the starch film from 4 MPa (without any reinforcing agent) to 6.7 MPa (with montmorillonite) and an increase in the mechanical strength by 50%. However, after the magnetization of montmorillonite, the mechanical strength of the magnetic montmorillonite/starch film decreases significantly but reaches an optimal value at 10%. This might be because the film formed using 10% magnetic montmorillonite and starch leads to a stable coexisting condition, which enhances the mechanical properties. However, the mass of Fe–O montmorillonite is much greater than those of Al–O and Si–O, increasing the magnetic material loads carried by starch and montmorillonite. This results in the degradation of the mechanical properties with an increase in the amount of magnetic montmorillonite. Thus, the tensile strength of the magnetic montmorillonite/starch film decreases. Analysis of the influence of the modification degree of montmorillonite on the properties of the starch nanocomposite membrane reveals that SDDACMt50, SODBACMt30 and SOACMt30 (SDDACMt50, starch/DDACMt50 film; SOACMt30, starch/OACMt30 film; SODBACMt50, starch/ ODBACMt50 film; DDACMt50, OMt modified with 50% DDAC) exhibit the highest tensile strengths of 6.22, 5.61 and 5.42 MPa, respectively. With modifier amounts of 30%–50%, SDDACMt, SODBACMt and SOACMt exhibited improved tensile strength and acceptable elongation at break, indicating that a moderate extent of montmorillonite modification could effectively improve the mechanical properties of the starch/montmorillonite nanocomposite films [23].

### 4.4. Infrared Spectral Analysis

**Figure 6:**
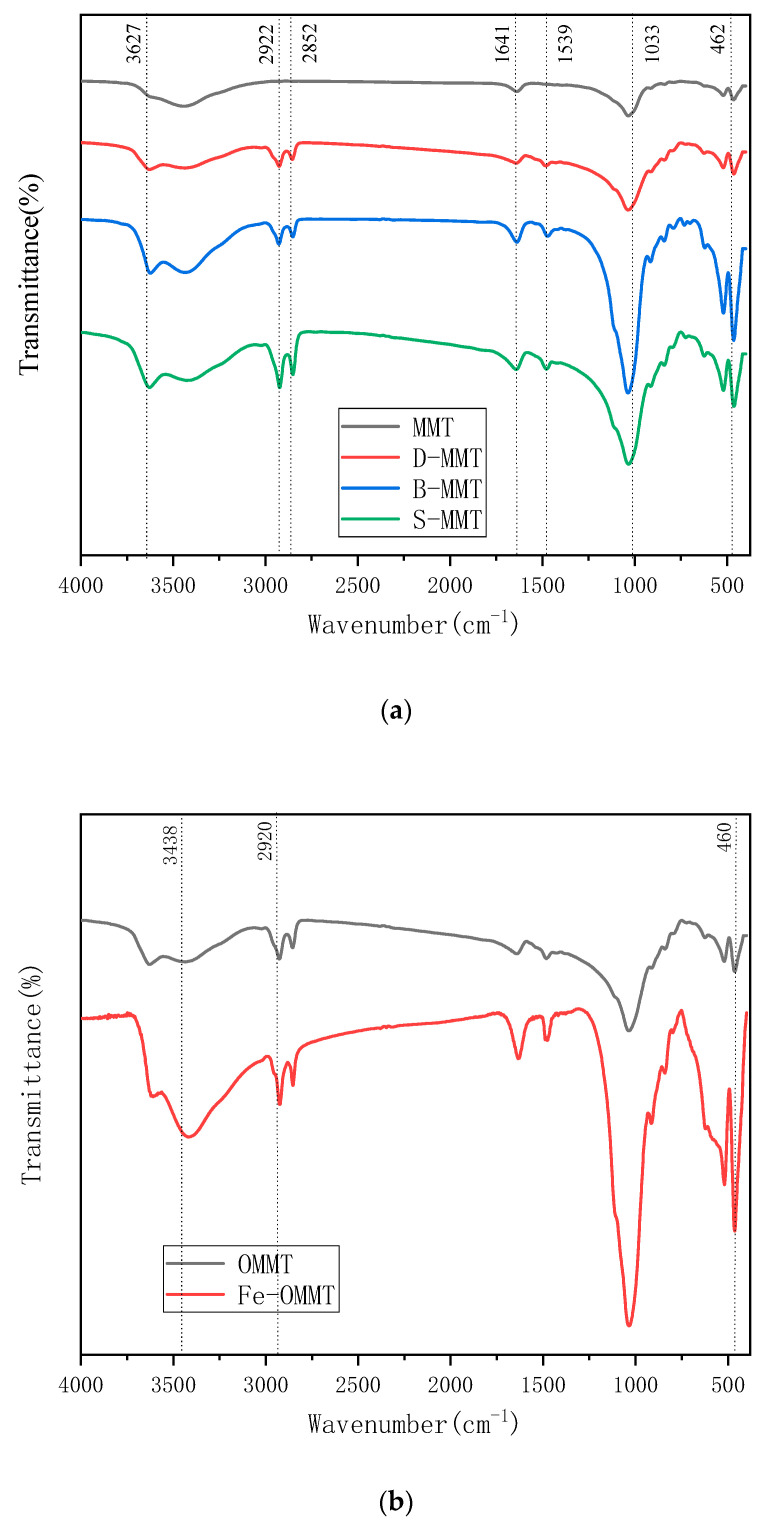
Fourier transform infrared (FTIR) spectra of (**a**) organic montmorillonite and (**b**) magnetic organic montmorillonite.

Figure 6 shows the Fourier transform infrared (FTIR) spectra depicting the changes in the chemical structure of montmorillonite after organic modification, to verify the presence of the organic modifier in the interlayer structure of montmorillonite. Figure 6a shows a band at 3627 cm^−1^ that arises from the hydroxyl vibrations in the montmorillonite and Si–O tetrahedra [24]. The FTIR peaks of trimethylamine appear at 2922 and 2852 cm^−1^. The vibration peak of –CH_2_– appears at 1033 cm^−1^, that of Cl^−^ appears at 1641 cm^−1^, and the characteristic peak of Al–O appears at 462 cm^−1^ [25,26] The organic carbon chains are thus successfully introduced by the organic modifier into montmorillonite and no other new functional groups are generated during the reaction. Figure 6b shows the FTIR spectrum of the magnetic montmorillonite powder, in which the peak intensity of the Fe-organically modified montmorillonite at 460 cm^−1^ has increased markedly due to the superposition of the characteristic Fe–O peak of the incorporated magnetic material, Fe_3_O_4_ and the characteristic peak of Al–O, indicating that the magnetic material is successfully introduced. Compared with the characteristic peak of the hydroxyl groups of hydrated montmorillonite at 3438 cm^−1^, the intensity of the –CH_2_– characteristic peak at 2920 cm^−1^ does not change markedly, indicating that no new functional groups are introduced. These characteristic peaks indicate that organic montmorillonite does not undergo any other reaction with iron(III) oxide and that magnetic montmorillonite powder is successfully obtained.

### 4.5. XRD Analysis

**Figure 7:**
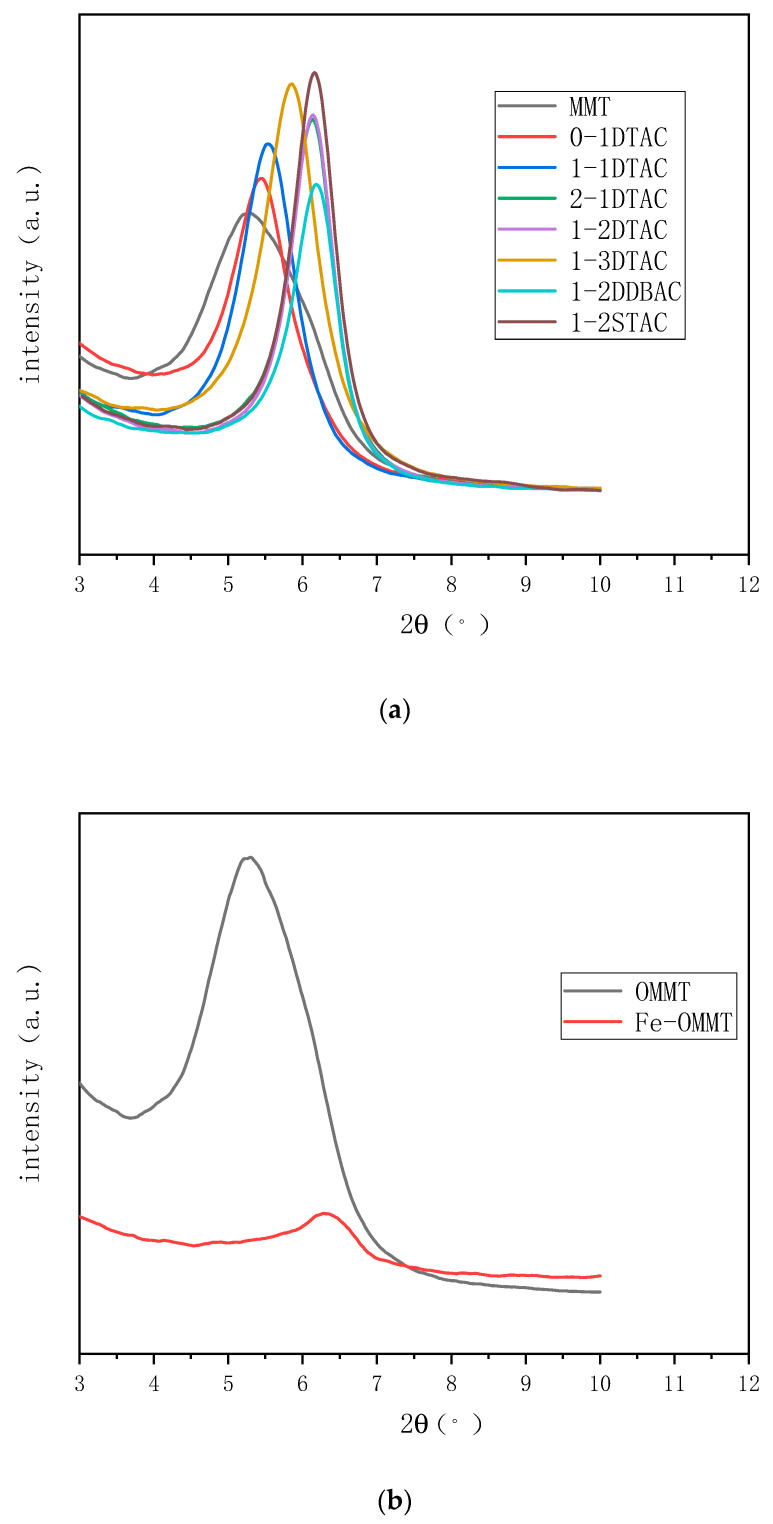
X-ray diffraction (XRD) patterns of (**a**) organic montmorillonite and (**b**) magnetic organic montmorillonite.

Table 1 shows the interlayer distance of organic montmorillonite prepared under different ultrasonic conditions, calculated using the Bragg’s law. Figure 7 shows the XRD patterns of organic montmorillonite prepared under different time and dosage conditions using different modifiers. Table 1 and Figure 7a reveal that montmorillonite shows the highest peak at 2*θ* = 5.28°; the interlayer distances are different for different samples. The highest peak value, which would represent the greatest distance between the layers of montmorillonite, is calculated from the change point using Prague’s law [27]. Figure 7a shows that the modified montmorillonite obtained under different conditions has different interlayer distances. The distance between the unmodified montmorillonite layers is small compared with those of 0-1 DTAC, 1-1 DTAC and 2-1 DTAC. Without ultrasonication, the change in the interlayer distance of montmorillonite is relatively small and only increases by 0.02 nm after 2 h of ultrasonication. The interlayer spacing of 2-1 DTAC reaches 1.49 nm, indicating that ultrasonication has a distinct effect on the interlayer exfoliation of montmorillonite layers. As the duration of ultrasonication increases, the interlayer exfoliation becomes more pronounced and the interlayer distance increases. Comparison among 1-1 DTAC, 1-2 DTAC and 1-3 DTAC indicates that increasing the modifier dosage increases the interlayer spacing. However, as complete ionic substitution is reached, the interlayer distance reaches a relatively equilibrated state, which is why the distances of 1-2 DTAC and 1-3 DTAC are almost identical. Comparison among 1-2 STAC, 1-2 DDBAC and 1-2 STAC indicates that with an increase in the length of the modifier carbon chain, the interlayer spacing of the montmorillonite interlayers increases; the layer distance of montmorillonite reaches a maximum in 1-2 STAC. Figure 7b shows the XRD pattern of organic montmorillonite, which exhibits a characteristic peak at 2*θ* = 5.15° and an interlayer distance of 0.14 nm. After magnetization, this peak shifts to 2*θ* = 6.18° and the interlayer distance increases nearly ten-fold to 1.49 nm [28,29]. Magnetization can evidently impart layer support to the montmorillonite to provide a stable interlayer support distance. The magnetization of montmorillonite and the enhanced interlayer distance of montmorillonite provide a foundation for further improvement in its barrier properties for use in biomass packaging materials. Hui Zhang et al. showed that the d-spacing of OMTs (organically modified montmorillonite) containing 10% modifiers was slightly higher than that of montmorillonite. The d-spacings of OMTs obtained with the addition of 30%, 50% and 70% modifiers were significantly higher than that of montmorillonite. With 30% DDAC and OAC, the d-spacings of the OMTs attained maximum values of 3.71 and 2.21 nm, respectively. These results indicated that with low amounts of modifiers, the alkyl chains in the interlayer space of the OMTs arranged themselves in the form of a monolayer parallel to the OMT surface layer [23].

### 4.6. Thermal Stability Analysis

**Figure 8:**
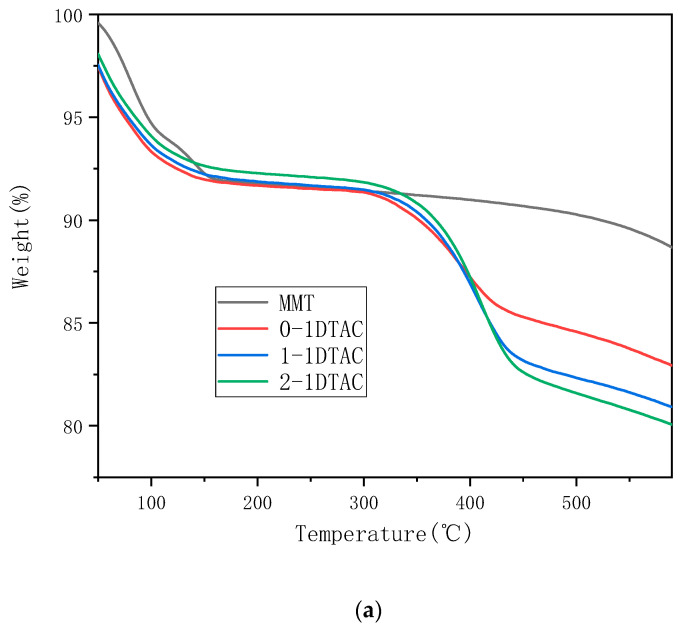

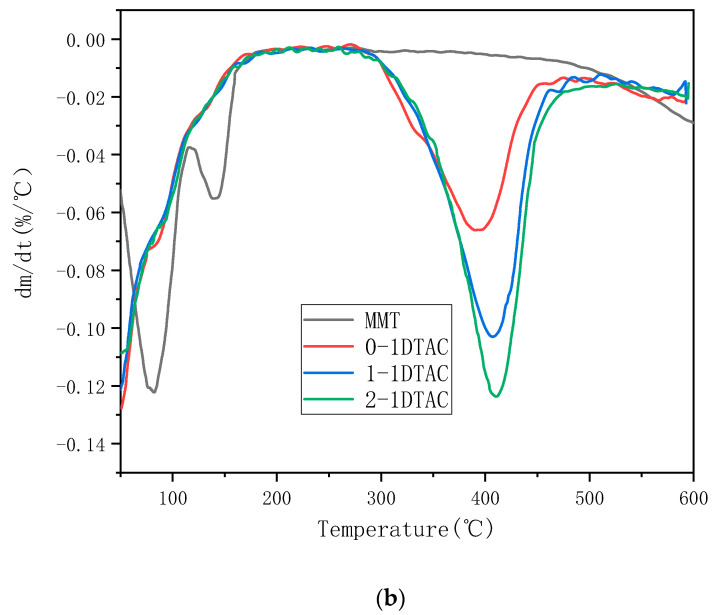
(**a**) TG (Thermogravimetry) and (**b**) DTG (derivative thermogravimetric)curves of montmorillonite samples prepared under different reaction times.

**Figure 9:**
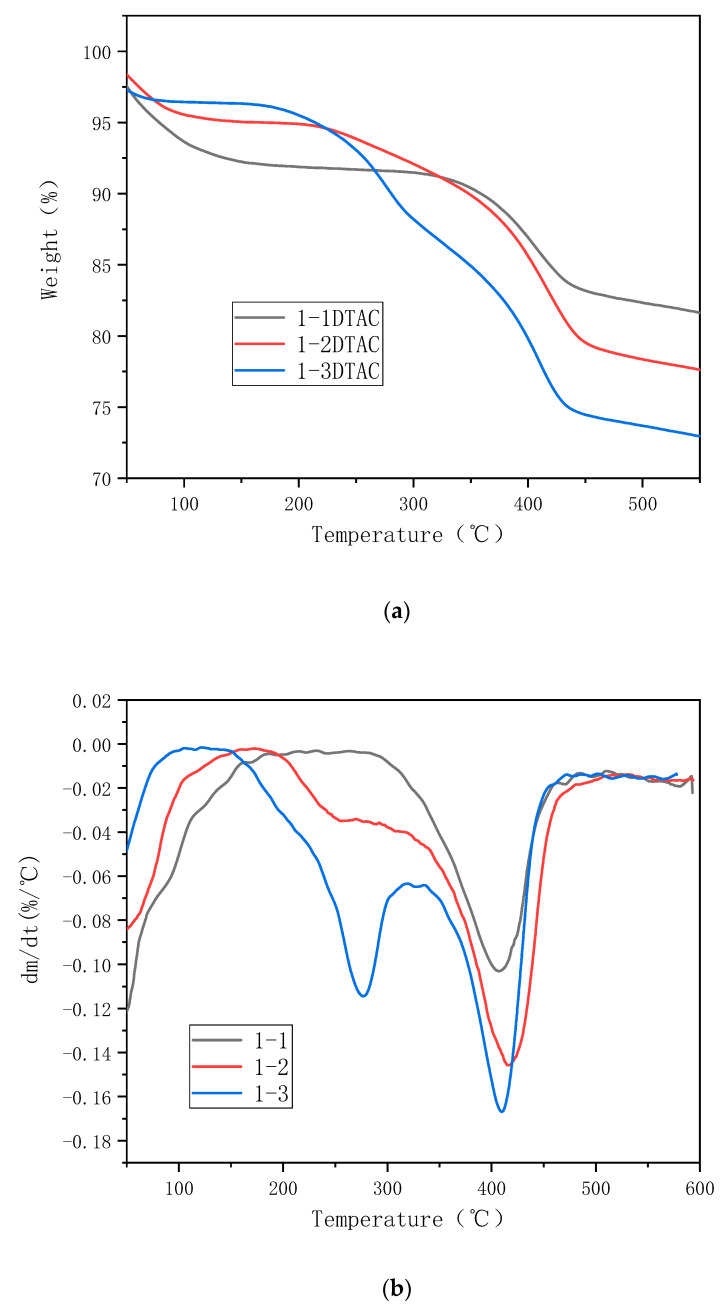
(**a**) TG and (**b**) DTG curves of montmorillonite samples treated with different modifier dosages.

**Figure 10:**
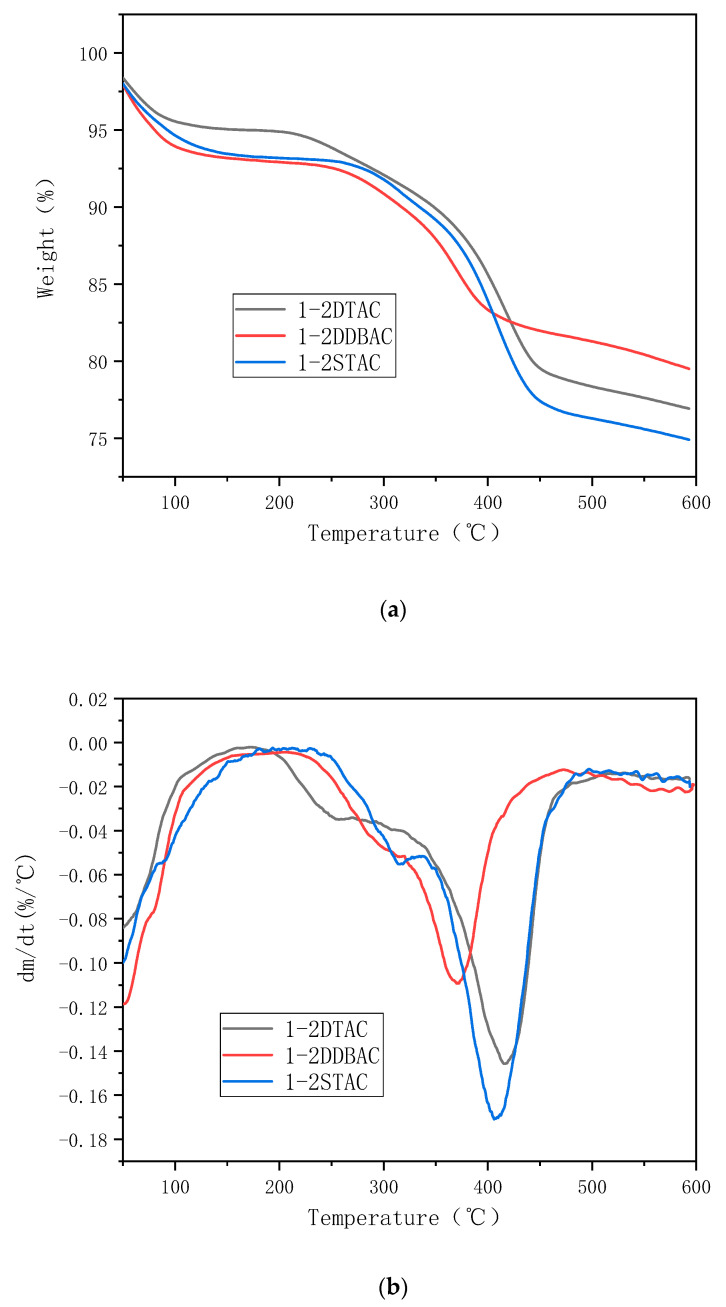
(**a**) TG and (**b**) DTG curves of 1-2 DTAC, 1-2 DDBAC and 1-2 STAC obtained by treating montmorillonite with different modifier types.

Figure 8, Figure 9 and Figure 10 present the thermal stability of organic montmorillonite obtained under different ultrasonication durations, modifier amounts and organic modifier types, respectively.

Figure 8 presents the TG (Thermogravimetry) and differential TG (DTG) curves of unmodified montmorillonite, 0-1 DTAC, 1-1 DTAC and 2-1 DTAC obtained under different ultrasonication durations. The weight loss of montmorillonite is marked at 150 °C, because the lamellar structure of montmorillonite can easily adsorb moisture from the air, which gradually evaporates and decomposes at high temperatures. Further, because montmorillonite is inorganic, the mass change is not distinct over this test range and the mass loss of unmodified montmorillonite is 11.7%. As shown in Figure 8, the remaining montmorillonite powders feature two weight loss ranges. The first weight loss range of 30–150 °C is due to the quality change caused by the separation of the water adsorbed in the sample. The second weight loss in the range of 300–500 °C is mainly due to the reaction of montmorillonite with the organic modifier—the –CH and –OH bonds and the amine group in the organic modifier are fully oxidized during the weight loss [30]. Further, 0-1 DTAC undergoes a surface-exchange reaction with the organic modifier without ultrasonication. The surface of montmorillonite adsorbs a large amount of the organic modifier; therefore, the interlayer distance does not improve but more mass is lost during thermal decomposition. The maximum decomposition temperature of this sample is 401 °C, which corresponds to a mass loss of 14.1%. The carbon chain of the organic modifier decomposes in the range of 300–500 °C; the maximum decomposition temperature of 1-1 DTAC is 405 °C with a mass loss of 19.1% and that of 2-1 DTAC is 408 °C with a mass loss of 19.6%. The effect of montmorillonite intercalation is markedly different under different reaction conditions. As the ultrasonication duration increases, the montmorillonite intercalation becomes more pronounced.

Figure 9 shows the TG and DTG curves of 1-1 DTAC, 1-2 DTAC and 1-3 DTAC obtained at different modifier dosages. The decomposition rate of 1-2 DTAC decreases at ~250 °C due to the separation of the organic matter from montmorillonite. The organic carbon chains of montmorillonite formed between montmorillonite and the modifier and the breakage of chemical bonds cause an endothermic reaction, resulting in a decreased weight loss rate; the maximum weight loss temperature of 1-2 DTAC is 418 °C, with a mass loss of 22.7%. Meanwhile, 1-3 DTAC has a more distinct endothermic peak at 260 °C. Addition of the organic modifier and hence, the endothermic peak formed by the breakage of –OH bonds, decomposes the sample into two parts. The maximum weight loss temperature of 1-3 DTAC is 420 °C and the mass loss is 25.9%.

Figure 10 shows the TG and DTG curves of 1-2 DTAC, 1-2 DDBAC and 1-2 STAC obtained by treating montmorillonite with different organic modifiers. All curves present a distinct endothermic peak at 250 °C. Because 1-2 DDBAC has a benzyl group, its decomposition temperature is inherently lower than those of the others; its maximum decomposition temperature is 376 °C with a mass loss of 20.1%. The maximum decomposition temperature of 1-2 STAC is 414 °C with a mass loss of 24.6%. Different kinds of modifiers can enhance the intercalation effect of montmorillonite and the longer the carbon chain, the more pronounced the effect.

### 4.7. Surface Hydrophobicity Characterization

**Figure 11:**
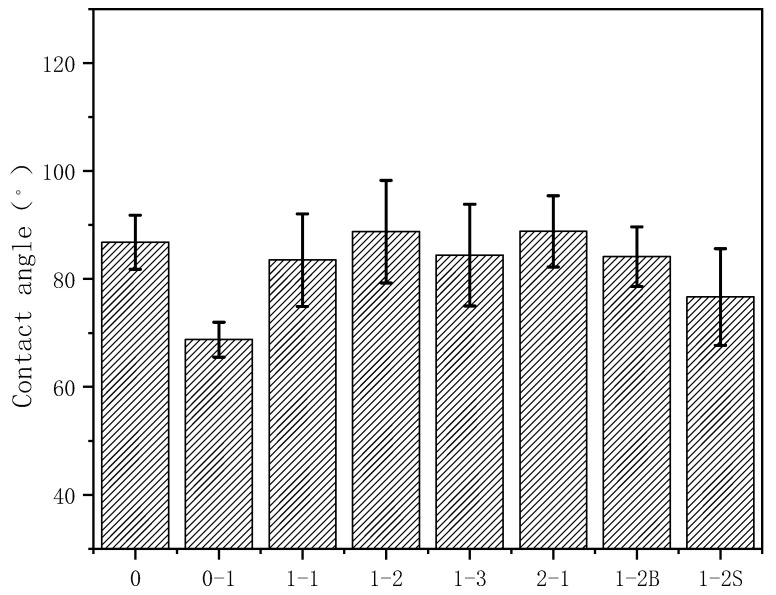
Contact angles of different montmorillonite/starch films.

Figure 11 shows the contact angles of the different montmorillonite/starch films. The contact angle of the starch film is 88°, indicating that it is hydrophilic. However, the contact angle of the 0-1 DTAC/starch film decreases and the hydrophobicity of the film is reduced after the addition of montmorillonite, because of the numerous −OH bonds of montmorillonite that readily bind to water, thereby reducing the water absorption ability of the montmorillonite/starch film [31]. As the amount of organic modifier increases, the hydrophobicity of the montmorillonite/starch film increases but some water molecules pass through the composite due to a continuous increase in the interlayer distance. The hydrophobicity of the montmorillonite/starch composite film therefore increases and then decreases and the contact angle of the montmorillonite/starch composite film is stable at 85° ± 5°. As the ultrasonication duration of the modification reaction increases, the hydrophobicity of the starch/montmorillonite composite film increases due to the increased amount of organic modifier intercalated in the montmorillonite and decreased number of −OH bonds, thus promoting hydrophobic effects.

## 5. Conclusions

This study explored the modification of montmorillonite with different ultrasonic conditions, organic modifiers and magnetization conditions. The intercalation reaction was employed as the main strategy to improve the barrier performance, while a magnetic material was used to control the paths for small molecules to pass through the material. The results reveal that due to the introduction of montmorillonite, the light transmittance of the composite film reduced by 10% at 600 nm. The maximum reduction in transmittance was observed in STAC-modified montmorillonite at 14.5%. The introduction of montmorillonite had a significant inhibitory effect on ultraviolet light, which was most apparent at 200 nm. The distance between the montmorillonite layers of the non-magnetized montmorillonite powder increased from 0.14 to 1.49 nm after magnetization. The mechanical properties of the non-magnetized organic montmorillonite/starch film increased from 5 to 6.5 MPa as the amount of montmorillonite increased; however, due to the introduction of Fe, the magnetized montmorillonite did not show improved mechanical properties. The oxygen permeability of the organic montmorillonite-modified starch film was lower (0.067 cm^3^/m^2^·d) than that of the non-magnetized montmorillonite starch film (0.097 cm^3^/m^2^·d) and the oxygen resistance capacity was close to zero. Magnetized modified montmorillonite and organic montmorillonite imparted evident oxygen-blocking ability when incorporated in starch films. In particular, the oxygen permeability of the ordered magnetic montmorillonite starch composite film increased from 0.23 to 0.113 cm^3^/m^2^·d with the increase in the additive amount in the ordered magnetic montmorillonite/starch composite films. This effect was stable and the oxygen-blocking ability reached the optimal value. However, the intercalation reaction of montmorillonite under other conditions was not clarified; the upper limit of the interlayer distance of montmorillonite was not clearly defined. We plan to explore whether the barrier properties of montmorillonite could be improved further with a further increase in the interlayer distance, in the future.

## Figures and Tables

**Table 1:** Spacing measurements of montmorillonite soil layers.

Sample	2*θ* (°)	Interlayer Spacing (nm)
MMT	5.28	0.16
0-1 DTAC	5.42	0.18
1-1 DTAC	5.54	0.21
2-1 DTAC	6.18	1.49
1-2 DTAC	6.12	0.95
1-3 DTAC	6.13	1.01
1-2 DDBAC	5.88	0.38
1-2STAC	6.16	1.25
